# Coronal and Root Canal Microbiota in Apical Periodontitis with Different PAI

**DOI:** 10.3390/microorganisms12081518

**Published:** 2024-07-24

**Authors:** Adelaide Teofani, Antonio Libonati, Valeria Unida, Silvia Biocca, Alessandro Desideri, Vincenzo Campanella

**Affiliations:** 1 Department of Biology, University of Rome Tor Vergata, Via della Ricerca Scientifica 1, 00133 Rome, Italy; adelaide.teofani@uniroma2.it (A.T.); desideri@uniroma2.it (A.D.); 2 Department of Clinical Sciences and Translational Medicine, University of Rome Tor Vergata, Via Montpellier, 00133 Rome, Italy; antonio.libonati@uniroma2.it; 3 Department of Systems Medicine, University of Rome Tor Vergata, Via Montpellier 1, 00133 Rome, Italy; valeria.unida@gmail.com (V.U.);

**Keywords:** endodontics, endodontic inflammation, microbiota, periapical periodontitis, 16S rRNA

## Abstract

Apical periodontitis is an inflammatory disease triggered by oral pathogens invading necrotic root canals. The aim of this study was to evaluate the coronal and root canal bacterial community profiles in primary endodontic infections with different periapical (PAI) indices in comparison to oral mucosa controls. A total of 31 patients with primary apical periodontitis, 14 with PAI-1 and 17 with PAI-3 were recruited. Microbial specimens from mucosa (control samples) and endodontic necrotic tissues were collected in each patient. Microbiota composition was studied through 16S ribosomal RNA gene amplicon sequencing analysis. Overall, 2953 taxa from 168 different genera of 451 various microbial species were retrieved in the controls and PAI-1 and PAI-3 groups. *Firmicutes* is the predominant phylum in the oral controls (34.5%) and PAI-1 (44.4%) groups, while *Bacteroidetes* is predominant in PAI-3 (38.6%). The *Proteobacteria* (21.5%) and *Fusobacteria* (12.5%) relative abundance is higher in oral controls while that of *Synergistetes* is higher in the PAI-1 (3.5%) and PAI-3 (2.5%) groups, being almost absent in controls (less than 0.1%). Most of the increased bacterial species found in the PAI groups were strict anaerobes. A diminished microbial diversity was found in apical periodontitis with higher PAI. These samples were also characterized by an increase in bacteria belonging to phyla and genera with an increased anaerobic character.

## 1. Introduction

Apical periodontitis (AP) is a bacterial infection of endodontic volume with a chronic immunoinflammatory reaction of all the apical supporting tissues supported by oral pathogens invading necrotic root canals [1]. Epidemiological studies in developed countries show that the frequency of AP in root-filled teeth and nontreated teeth was 39% in the general population [2] and the World Health Organization describes AP as a pandemic disease [3]. The histopathological aspect of AP is characterized by the prevalence of macrophages, mononuclear cells, lymphocytes, and plasma cell types [4]. The relationship between the severity of the AP and the inflammatory reaction could be linked to a specific microbial ecology. The role of bacteria in the etiology of apical periodontitis was first demonstrated by Kakehashi and colleagues [5] who used germ-free and conventional laboratory rats to compare the inflammatory reactions in surgically exposed dental pulps. Whereas no apical periodontitis was detected in germ-free rat, all conventional laboratory rats developed a pulpal necrosis associated with a severe inflammatory reaction around periapical tissues [5]. Many studies support the idea of natural selection within necrotic root canals, influencing both the synergistic and antagonistic actions of colonizing pathogens [6,7]. Strictly anaerobic bacteria prevalently compose the microbial population of untreated necrotic root canals, with species mostly belonging to the following genera: *Peptostreptococcus*, *Prevotella*, *Porphyromonas*, *Fusobacterium*, *Eubacterium*, and *Actinomyces*. The presence of these pathogens in necrotic root canals correlates with the observation of the presence of apical inflammatory lesions in intraoral radiographs [8,9,10].

Primary AP associated with pulp necrosis usually heals after a root canal treatment that combines a chemo-mechanical debridement of infected tissues with a root canal filling [11]. Unsuccessfully treated root canals may exhibit a persistent inflammation known as secondary apical periodontitis, which have a different microbial composition from those of untreated roots. In these areas, culture-based studies usually identified Gram-positive facultative anaerobic genera, such as Streptococcus, Lactobacillus, and Enterococcus [12]. Clinical studies underlie how a great proportion of root-filled teeth shows radiographic evidence of secondary AP and the association of apical periodontitis with several systemic diseases, including cardiovascular disease, diabetes mellitus, liver disease and blood disorders [13]. Before the implementation of high-throughput sequencing (HTS) technologies the knowledge regarding microbiota composition in different clinical situations was limited to those species that could be cultured in laboratory. The use of both culture-dependent and independent methodologies to investigate the microbiota in root-filled teeth, particularly those associated with peri-radicular lesions, yielded to different results. These findings underscored the benefits of utilizing HTS to assess microbial diversity in apical periodontitis, preventing the underestimation of the role played by uncultivated species in the etiology of AP [14,15]. The HTS technologies and, in particular the 16S ribosomal RNA (rRNA) gene sequencing, supply an effective mean to fully characterize the microbiota of the oral cavity [16]. This technology allowed to identify taxa still undiscovered in oral microbiota showing that the bacterial diversity in the oral cavity is greater than expected [17]. A deep knowledge of the microbiota with different periapical (PAI) indices is still missing.

In this study, we have investigated the bacterial community profiles of endodontic microbiota in primary infections using 16S rRNA gene amplicon sequencing comparing microbiota of healthy oral controls with microbiota of primary infections with a periapical index (PAI) score 1 and a PAI ≥ 3. PAI provides an ordinal scale of 5 scores ranging from 1 (absence of apical radiolucency) to 5 (severe periodontitis with exacerbating radiographic features) and it is based on the use of reference radiographs of teeth with verified histological diagnoses [18]. Our double aim was to identify differences between healthy and differently affected samples and between samples coming from an environment with a different PAI. We find a diminished microbial diversity in the apical periodontitis samples in comparison to the oral controls and the presence of bacteria belonging to phyla and genera with an increased anaerobic character that increases with the severity of the disease.

## 2. Materials and Methods

### 2.1. Recruitment of Patients and Clinical Characteristics

Patients with a clinical and radiographic confirmed diagnosis of periodontitis and confirmed pulp necrosis by cold viability test supported by cavity test were prospectively recruited between April 2022 and April 2023 at the university hospital (PTV, University of Rome Tor Vergata). Our sampling included 31 patients (16 female and 15 male) affected by primary AP, of which 14 with a PAI = 1 (PAI-1) and 17 with a PAI ≥ 3 (PAI-3) in teeth affected by necrotic tissues, as detected and defined by periapical radiograph. To mitigate inter-subject variability, we collected paired samples from oral mucosa and endodontic lesioned tissues in each patient. The teeth selected were subjected to a strict disinfection procedure. In detail, oral cavity was disinfected for at least 30 s rinsing the mouth with 0.2% chlorhexidine solution; a rubber dam with clamp and the tooth to be treated were disinfected with 3% hydrogen peroxide and 2% chlorhexidine; pulp chamber was disinfected with 2% chlorhexidine placed with cotton ball for 30 s and rinsed with sterile saline solution. The microbiological samples were collected from pulp chambers with sterile paper point size #20 ISO. In order to loosen the biofilm from the root canal walls, a file (FF) ISO size #15 (type S-file) was introduced to the root canal. Sterile saline solution was introduced into the canal with a syringe and endodontic needle, avoiding root canal overfilling. A sample from the root canal was collected with paper point size #20. For all patients a sample from oral mucosa was collected by a sterile cotton pellet, as a control.

Specimens were collected in vials of “Swab Collection and DNA Preservation System (Bulk Format)”, NORGEN Bioteck corp. (Thorold, ON, Canada).

### 2.2. DNA Extraction Quality Control

DNA extraction from the stool samples was performed with PSP Spin Stool DNA Kit Plus (Invitek Molecular, Berlin, Germany), as previously described [19]. The purified DNA was quantified using a NanoDrop spectrophotometer ND1000 (Termofisher Waltham, MA, USA).

### 2.3. Sequencing, Raw Data Processing Taxonomic Assignment and Statistical Analysis

16S rRNA amplicon (V3–V4 regions) sequencing analysis was performed with an Illumina MiSeq (San Diego, CA, USA) utilizing a 2 × 300 bp configuration. To ensure data quality, raw sequencing data underwent rigorous quality control and filtering steps for removal of substandard reads, adapter sequences, and PCR artifacts. To prepare the data, Cutadapt [20] was employed to remove primers and adapters. Quality assessment was executed through FastQC, and low-quality bases were pruned (trimmed) using the FastP tool [21]. Subsequently, the preprocessed reads were analyzed utilizing the QIIME2 pipeline [22]. This entailed chimaera-checking reads and their clustering into amplicon sequence variants (ASVs) using the DADA2 algorithm [23]. All data manipulations and statistical analyses were conducted within the R environment (version 3.6), utilizing the vegan 2.5.6 [24] and phyloseq 1.30.0 [25] packages. Normalization of sample data was performed using the DESeq2 R [26]. ASVs underwent filtration with only those present in a minimum of 10% of samples being considered. To assign taxonomic classifications to representative sequences obtained from DADA2, the q2-feature-classifier and the Human Oral Microbiome Database (HOMD) [27] were employed. After eliminating low-frequency ASVs and applying data normalization with DESeq2, we quantified species-level α-diversity through three metrics: Chao1, Shannon, and Simpson. β-diversity was assessed using four metrics, Bray–Curtis, weighted, unweighted, and generalized Unifrac, and was represented using the Principal Coordinates Analysis (PCoA), a multivariate statistical technique used for visualizing similarities or dissimilarities between data points. To identify differential abundant taxa across the groups, we used the negative binomial model included in the DESeq2 package. The tax_glom function within the phyloseq package was utilized to aggregate ASVs read counts across various taxonomic levels, facilitating comparisons between samples. Resulted *p*-values have been corrected for multiple tests by Deseq2 using the Benjamini–Hochberg correction. We considered as statistically significant only differences in abundance with an adjusted *p*-value < 0.05.

## 3. Results

### 3.1. Sequencing Data and Diversity in Oral Microbiota

Our study involved 62 samples, including 31 patient’s oral microbiota used as oral controls (OC) and 31 diseased tissues, diversified in 14 necrotic tissues (PAI-1) and 17 periapical lesions (PAI-3). Oral controls are samples taken from the oral mucosa of the patients that are not affected by periodontal disease and/or endo-perio lesions, but only by local pulp necrosis and apical periodontitis, with different PAI. Oral mucosa is then taken as an indicator of the oral microbiota in a healthy environment of patients locally affected by apical periodontitis.

Using the QIIME2 (Quantitative Insights Into Microbial Ecology) pipeline, we identified a total of 5198 different amplicon sequence variants (ASVs) of which 2964 passed the filtering steps described in MM.

**Figure 1:**
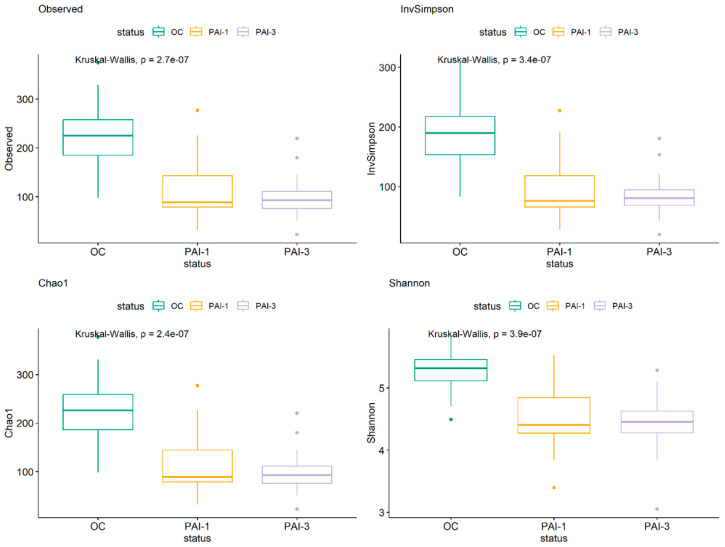
α-diversity plots to visualize the difference of microbiota structure between the OC, PAI-1, and PAI-3 groups, according to four indices: observed species, Inverse Simpson, Chao1 and Shannon. Green: OC, orange: PAI-1 and violet: PAI-3.

**Figure 2:**
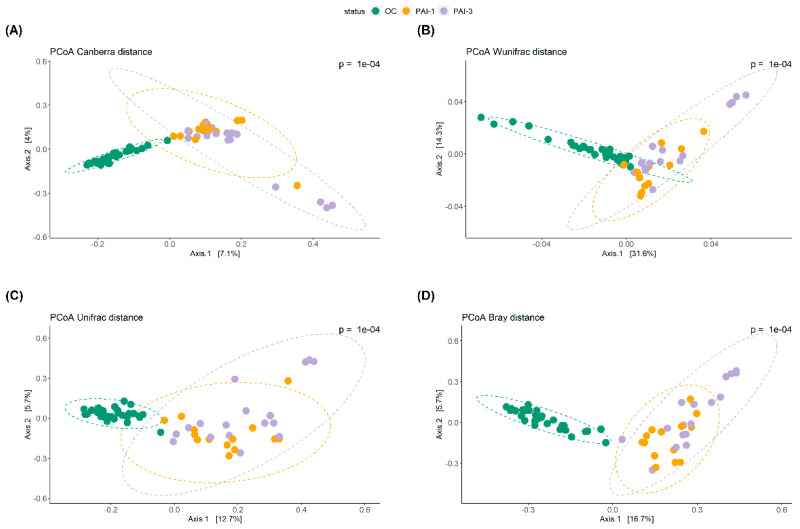
Principal Coordinate Analysis (PCoA) plots are used for (**A**) Canberra, (**B**) Weighted Unifrac (Wunifrac), (**C**) Unifrac and (**D**) Bray–Curtis (Bray) metrics. Each dot represents an individual sample. PCoA plots show dimensions with the highest differences and normal confidence ellipsoids for the sample sets. Green: OC, orange: PAI-1 and violet: PAI-3.

Figure 1 shows the α-diversity of the OC, PAI-1 and PAI-3 groups whose difference was statistically significant for all the metrics (*p* = $2e^{-6}$ observed, *p* = $2.4e^{-6}$ Chao1, *p* = $1.6e^{-6}$ Shannon and *p* = $2.3e^{-6}\;$ Simpson), indicating a diminished microbial diversity in lesioned tissues (PAI-1 and PAI-3) and a strong association between the loss of oral microbiota diversity and the progression of periodontitis, as previously reported [28,29]. The β-diversity, shown in Figure 2, was analyzed by the Principal Coordinate Analysis (PCoA) method computed using the four metrics Canberra, Weighted Unifrac (Wunifrac), Unifrac and Bray–Curtis (Bray). A distinct separation between healthy OC and PAI-1 and PAI-3 samples is confirmed, with a definite variation in microbial structure and composition. The differences between PAI-1 and PAI-3 samples are less noticeable.

### 3.2. Differential Analysis Abundance of OC, PAI-1 and PAI-3 Phyla

**Figure 3:**
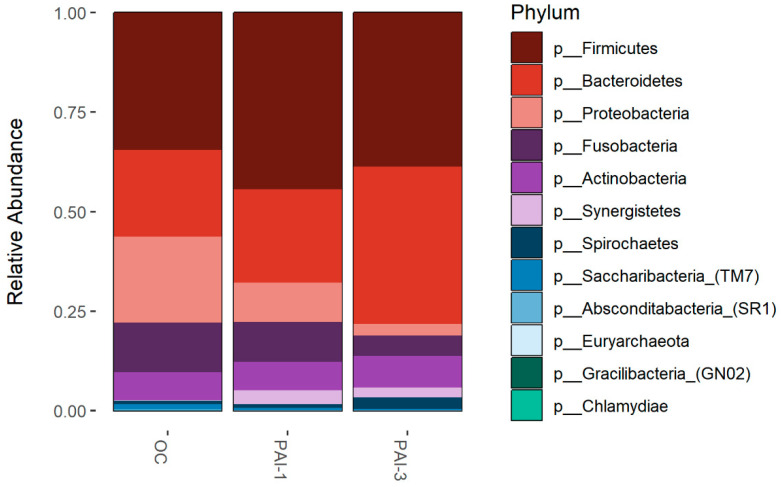
Bar-plots of relative abundance at phylum level, identified in oral microbiota of the OC, PAI-1 and PAI-3 groups. The x-axis represents groups and the y-axis the relative abundance presented as the proportion of the species in that group. Different colored bars are different species, and the length of the bar represents the size of the proportion of the species.

The relative abundance of the predominant phyla in OC, PAI-1 and PAI-3, reported in Figure 3, was evaluated by agglomerating the taxa at Phylum level. *Firmicutes* is the predominant phylum in the OC (34.5%) and PAI-1 (43.35%) groups, while *Bacteroidetes* is predominant in PAI-3 (39.6%). The *Proteobacteria* and *Fusobacteria* relative abundance is higher in OC while that of *Synergistetes* is higher in the PAI-1 (3.5%) and PAI-3 (2.5%) groups and almost absent in OC (less than 0.1%). *Actinobacteria* appears to be comparable among the three groups.

**Figure 4:**
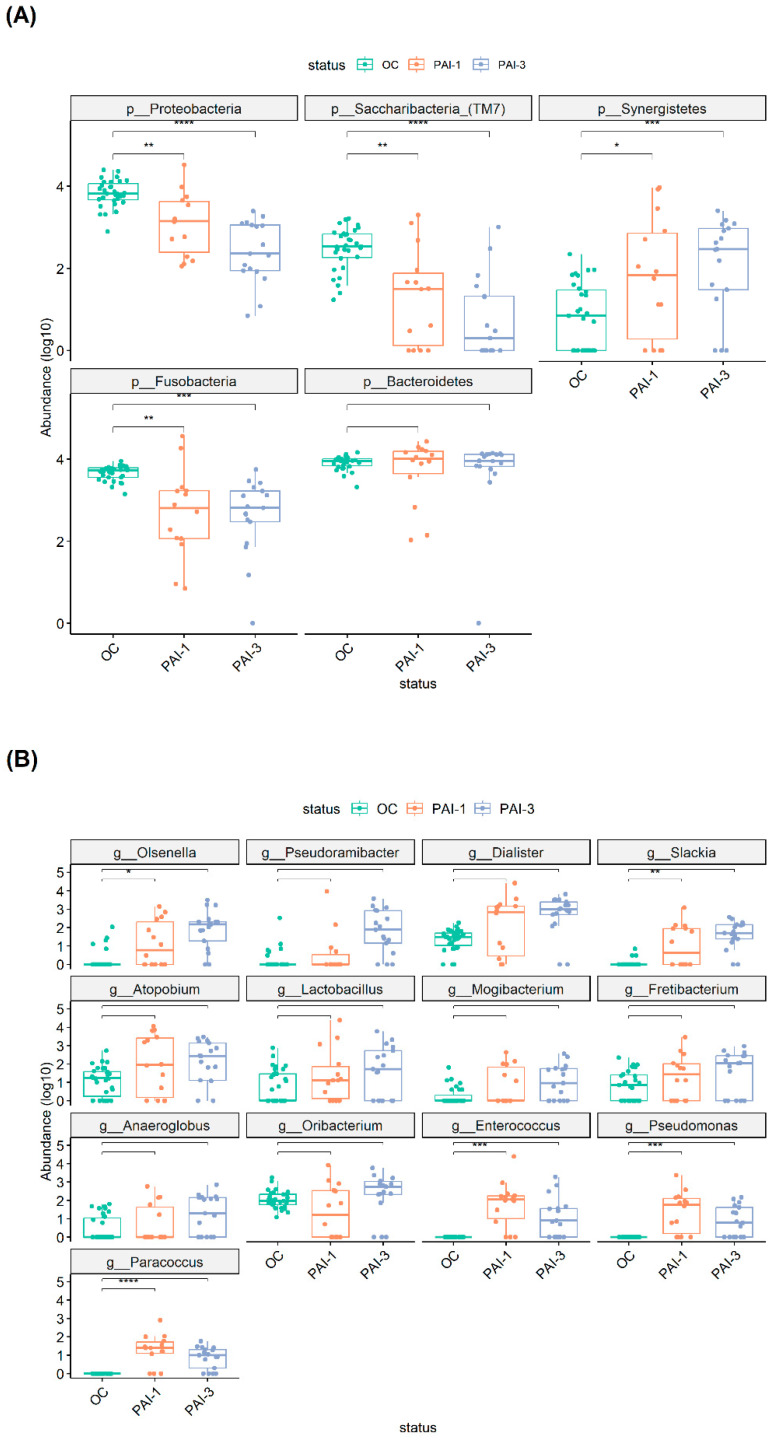
Boxplots representing the differential abundant phyla (**A**) and genera (**B**), among the OC, PAI-1 and PAI-3 groups. The relative abundance is plotted in log10 on the y-axis. Asterix indicates statistically significant comparisons. (* *p* < 0.05, ** *p* < 0.01, *** *p* < 0.001, **** *p* < 0.0001). Green: OC, orange: PAI-1 and violet: PAI-3.

Further analysis of the difference in microbial composition between healthy and lesioned tissues was carried out using a negative binomial generalized linear model, conducting paired tests to compare microbial abundances between OC and PAI-1, as well as the OC and PAI-3 groups. We also assessed differential abundance between the PAI-1 and PAI-3 groups, examining various taxonomic levels (Figure 4A). A statistically significant declining trend in the *Proteobacteria* and *Saccharibacteria* phyla going from OC to PAI-3 is highlighted. Conversely, the percentage of *Synergistetes* is increasing from OC to PAI-1/PAI-3 (Figure 4A).

### 3.3. Comparisons of Microbiota in Apical Periodontitis with Different PAI at the Genera and Species Levels

At the genera level, the comparisons OC vs. PAI-3, OC vs. PAI-1 and PAI-1 vs. PAI-3 are shown in Supplementary Table S1. The OC and PAI-3 groups comparison identified 49 genera exhibiting statistically significant differential abundance (padj < 0.05). Among these, 13 showed an increased abundance and 36 a reduced abundance in the PAI-3 group. Comparison of the OC and PAI-1 samples identified 39 statistically significant differentially abundant genera with 10 genera having an increased abundance in PAI-1. Of note, the genera *Atopobium*, *Lactobacillus*, *Fretibacterium*, *Peptostreptococcaceae*_[XI][G-6], and *Peptostreptococcaceae*_[XI][G-1] exhibited an increased abundance in OC when compared to both PAI-1 and PAI-3 samples. When comparing the PAI-1 and PAI-3 groups, a disparity in the abundance of the *Pseudoramibacter* and *Peptostreptococcaceae*_[XI][G-3] genera emerged, denoting an enhanced presence in the PAI-3 group (Table S1).

Figure 4B shows only the genera that display a statistically significant increase in PAI-1 or in PAI-3 or in both in comparison to the OC group.

Analysis at the species level is shown in Table 1 and it reveals statistically significant differential abundances (padj < 0.05) between the three groups. We identified an increase in *Pseudoramibacter alactolyticus* abundance within the PAI-3 group. The significant increase in this bacterium in the periapical root is in line with its anaerobic nature. In contrast, *Capnocytophaga sputigena* decreased in abundance both in the PAI-1 and PAI-3 groups when compared with OC (Table 1).

Additionally, when we compared species abundances in PAI-1 vs. OC samples, we noted a statistically significant increase in abundance of *Dialister invisus*, *Prevotella oralis*, *Paracoccus yeei*, *and Enterococcus casseliflavus* (Table 1). Notably, several species exhibited significant alterations only in the OC vs. PAI-3 comparison, but not in the OC vs. PAI-1 comparison. This is the case for *Peptostreptococcaceae* [XI][G-1], [*Eubacterium]_infirmum*, *Dialister pneumaosintes*, *Prevotella baroniae*, *Fretibacterium fastidiosum*, *Oribacterium* sp._HMT_102, *Anaeroglobus geminatus*, *Slackia exigua*, *Olsenella uli* and *Bacteroidaceae_*[G-1] *bacterium*_HMT_272. On the other hand, the abundance of *D. invisus* and *P. yeii* was significantly elevated in both PAI-1 and PAI-3 when compared to OC (Table 1). Among the species that increased only in the OC vs. PAI-3 comparison, there are: *A. geminatus*, which is an obligate anaerobe species and *D. pneumaosintes*, *P. baroniae*, *F. fastidiosum*, *S. exigua* and *O. uli*, that are all anaerobic, microaerophilic, or facultatively anaerobic species commonly found in the context of periodontitis and, specifically, in periapical root canal infection [29,30,31].

## 4. Discussion

Bacterial microbiota of apical periodontitis showed distinctive changes in comparison to oral mucosa controls, showing two main distinct features: (1) a reduced microbiota diversity in PAI-1 and PAI-3 samples in comparison to the oral controls and (2) an increased trend from PAI-1 to PAI-3 toward the anaerobic species, independently from the relatively large inter-individual variability present in patients.

The reduced microbiota diversity is highlighted by the comparison of the α-diversity values, which shows a statistically significant difference for all the metrics (*p* = $2e^{-6}$ observed, *p* = $2.4e^{-6}$ Chao1, *p* = $1.6e^{-6}\;$ Shannon and *p* = $2.3e^{-6}$ Simpson). In line, a strong association between the loss of oral microbiota diversity and the progression of periodontitis has been reported [32]. Recently, it has been also reported that the decrease in microbiota diversity correlates with the increase in the severity of apical periodontitis [28]. Here, we find a similar trend although, in our case, the differences between PAI-1 and PAI-3 are not statistically significative.

For instance, at the Phylum level statistically significant declining trend is observed in the *Proteobacteria* and *Saccharibacteria* going from OC to PAI-3 with PAI-1 having an intermediate value. Conversely, the percentage of *Synergistetes* is increasing going from OC to PAI-1/PAI-3 (Figure 4). In line, members of the phylum *Synergistetes* have been proposed to be linked to periodontal disease [33,34]. *Fusobacteria* are higher in OC with respect to the PAI-1 and PAI-3 groups. Interestingly, *Firmicutes*, *Actinobacteria*, *Bacteroidetes*, *Proteobacteria* and *Fusobacteria* were described present in endodontic infections. Our study confirms the higher abundance of *Bacteroidetes* in larger lesions (PAI-3) and the predominance of *Synergistetes* phylum, while *Firmicutes*, *Proteobacteria* and *Fusobacteria* are abundant in smaller ones (PAI-1) [35,36].

Also at the genera level, we notice an increase in the anaerobic or microaerophilic genera, such as *Lactobacillus*, *Fretibacterium*, *Dialister*, *Slackia*, *Anaeroglobus* and *Pseudoramicater* going from controls OC to lesioned PAI-1/PAI-3 samples., in line with previously described analysis [1,37,38,39].

The analysis at the species level, also show an increase in the anaerobic species. We identified an increase in *Pseudoramibacter alactolyticus* abundance within the PAI-3 group. Notably, *P. alactolyticus* is an anaerobic, lactic acid bacterium, which has been found to be common and more abundant in endodontic infections, peri-implantitis, and diseased implants compared to healthy periodontal tissue [40]. An interesting finding concerns the decrease in *Capnocytophaga sputigena* both in the PAI-1 and PAI-3 groups when compared with OC (Table 1). This is a slow-growing, capnophilic (carbon dioxide-requiring), facultatively anaerobic, Gram-negative bacterium, which has been identified as part of the 4th complex in subgingival plaque associated with periodontitis [41,42,43,44]. *C. sputigena*, along with other periodontal pathogens such as *Porphyromonas gingivalis and Treponema denticola*, can interfere with host defense mechanisms and contribute to the pathogenesis of periodontitis [44]. The decrease in *C. sputigena*, that occurs together with the shift in the PAI-3 environment, could agree with the facultative anaerobic nature of this species. Interestingly, the differences in *C. sputigena* abundances are significant also within the same subject.

In conclusion, bacterial microbiota of AP showed an increased trend from PAI-1 to PAI-3 toward the anaerobic species, together with a reduced diversity. In line, it has been recently reported an increase in anaerobic genera, related to size of AP lesion [1,35,45]. Notably, AP has heterogenous etiology in which different species combinations can lead to the same disease outcome (PAI-1 or PAI-3). One possible explanation is that the origin of AP is related to the bacterial products expressed by various species organized in different consortia, that may play an important role in the community physiology and pathogenicity. It is important to note that the phases and microbial composition of periodontal infections can vary among individuals and may be influenced by factors such as disease severity and host immune response [46]. The understanding of these phases and their associated microbial complexes provides insights into the pathogenesis of periodontal disease and may guide the development of targeted therapeutic strategies. In the future, more in-depth results may be achieved analyzing shotgun sequences of the full microbiota, that will permit to achieve information on the functional profile of the analyzed communities.

## Figures and Tables

**Table 1:** Statistically significant (padj < 0.05) species in the three comparisons: OC vs. PAI-3, OC vs. PAI-1 and PAI-1 vs. PAI-3.

Species	OC vs. PAI-3	OC vs. PAI-1	PAI-1 vs. PAI-3
*Fusobacterium periodonticum*	↓ ^1^	↓	-
*Prevotella melaninogenica*	↓	↓	-
*Haemophilus parainfluenzae*	↓	↓	-
*Granulicatella adiacens*	↓	↓	-
*Porphyromonas pasteri*	↓	↓	-
*Leptotrichia* sp. *HMT_215*	↓	↓	-
*Rothia mucilaginosa*	↓	↓	-
*Prevotella nanceiensis*	↓	↓	-
*Campylobacter concisus*	↓	↓	-
*Gemella sanguinis*	↓	↓	-
*Streptococcus parasanguinis_clade_411*	↓	↓	-
*Bergeyella* sp.*_HMT_322*	↓	↓	-
*Leptotrichia hongkongensis*	↓	↓	-
*Saccharibacteria_(TM7)_[G-1] bacterium_HMT_352*	↓	-	-
*Rothia dentocariosa*	↓	↓	-
*Lachnoanaerobaculum umeaense*	↓	↓	-
*Fusobacterium nucleatum_*subsp. *vincentii*	↓	↓	-
*Corynebacterium matruchotii*	↓	↓	-
*Capnocytophaga gingivalis*	↓	↓	-
*Capnocytophaga leadbetteri*	↓	↓	-
*Veillonella atypica*	↓	↓	-
*Lautropia mirabilis*	↓	↓	-
*Prevotella intermedia*	↓	↓	-
*Streptococcus salivarius*	↓	↓	-
*Dialister invisus*	↑	↑	-
*Veillonella dispar*	↓	↓	-
*Slackia exigua*	↑	-	-
*Prevotella salivae*	↓	↓	-
*Atopobium parvulum*	↓	-	-
*Pseudoramibacter alactolyticus*	↑	-	↑
*Prevotella nigrescens*	↓	↓	-
*Prevotella pallens*	↓	↓	-
*Saccharibacteria_(TM7)_[G-3] bacterium_HMT_351*	↓	-	-
*Veillonella rogosae*	↓	-	-
*Ruminococcaceae_[G-2] bacterium_HMT_085*	↓	-	-
*Olsenella uli*	↑	-	-
*Gemella morbillorum*	↓	-	-
*Capnocytophaga* sp. *HMT_326*	↓	-	-
*Alloprevotella* sp. *HMT_308*	↓	-	-
*Bacteroidales_[G-2] bacterium_HMT_274*	↓	-	-
*Bacteroidaceae_[G-1] bacterium_HMT_272*	↑	-	-
*Veillonella parvula*	↓	↓	-
*Stomatobaculum* sp. *HMT_097*	↓	-	-
*Ruminococcaceae_[G-1] bacterium_HMT_075*	↓	↓	-
*Megasphaera micronuciformis*	↓	-	-
*Prevotella* sp. *HMT_317*	↓	↓	-
*Schaalia* sp. *HMT_180*	↓	↓	-
*Saccharibacteria_(TM7)_[G-5] bacterium_HMT_356*	↓	-	-
*Capnocytophaga sputigena*	↓	↓	↓
*Catonella morbi*	↓	-	-
*Fretibacterium fastidiosum*	↑	-	-
*Rothia aeria*	↓	-	-
*Cardiobacterium hominis*	↓	↓	-
*Oribacterium* sp. *HMT_102*	↑	-	-
*Capnocytophaga granulosa*	↓	↓	-
*Granulicatella elegans*	↓	-	-
*Kingella oralis*	↓	↓	-
*Selenomonas sputigena*	↓	-	-
*Haemophilus sputorum*	↓	-	-
*Prevotella* sp. *HMT_396*	↓	-	-
*Campylobacter gracilis*	↓	↓	-
*Tannerella forsythia*	↓	↓	-
*Leptotrichia* sp.*_HMT_417*	↓	-	-
*Corynebacterium durum*	↓	-	-
*Prevotella denticola*	↓	-	-
*Porphyromonas endodontalis*	↓	-	-
*Peptostreptococcaceae_[XI][G-1] [Eubacterium]_infirmum*	↑	-	-
*Abiotrophia defectiva*	↓	-	-
*Alloprevotella* sp.*_HMT_914*	↓	-	-
*Treponema denticola*	↓	-	-
*Haemophilus* sp.*_HMT_036*	↓	↓	-
*Lachnospiraceae_[G-3] bacterium_HMT_100*	↓	-	-
*Alloprevotella* sp.*_HMT_473*	↓	-	-
*Tannerella* sp.*_HMT_286*	↓	↓	-
*Actinomyces graevenitzii*	↓	-	-
*Dialister pneumosintes*	↑	-	-
*Prevotella baroniae*	↑	-	-
*Paracoccus yeei*	↑	↑	-
*Neisseria elongata*	↓	-	-
*Filifactor alocis*	↓	-	-
*Prevotella* sp.*_HMT_300*	↓	-	-
*Prevotella oulorum*	↓	↓	-
*Bacteroidetes_[G-5] bacterium_HMT_511*	↓	-	-
*Anaeroglobus geminatus*	↑	-	-
*Mycoplasma faucium*	↓	-	-
*Selenomonas noxia*	-	↓	-
*Enterococcus casseliflavus*	-	↑	-

^1^ The ↓ indicates a significant decreased abundance, while ↑ indicates a significant increased abundance in the second group in comparison with the first one.

## Data Availability

The raw data supporting the conclusions of this article was submitted on SRA (Bioproject PRJNA1124083) and will be made available by the authors on request.

## Supplementary Materials

The following supporting information can be downloaded at: https://www.mdpi.com/article/10.3390/microorganisms12081518/s1, Table S1: Statistically significant genera in the three comparisons.
