# Supplementary material for: Coronal and Root Canal Microbiota in Apical Periodontitis with Different PAI
Source: Microorganisms. 2024 Jul 24;12(8):1518. doi: 10.3390/microorganisms12081518 (PMC11356064; doi:10.3390/microorganisms12081518)
Supplement: Supplementary file 1 [file microorganisms-12-01518-s001.zip › microorganisms-3080841-supplementary.pdf]

Supplementary Material

Coronal and root canal microbiota in apical periodontitis with different PAI index

Adelaide Teofani[1], Antonio Libonati[2], Valeria Unida[3], Silvia Biocca[3], Alessandro Desideri[1] and Vincenzo Campanella[2*]

[1] Department of Biology, University of Rome Tor Vergata, Via della Ricerca Scientifica 1, 00133, Rome, Italy.

[2] Department of Clinical Sciences and Translational Medicine, University of Rome Tor Vergata, Via Montpellier, 00133 Rome, Italy.

[3] Department of Systems Medicine, University of Rome Tor Vergata, Via Montpellier 1, 00133, Rome, Italy.

* Correspondence: vincenzo.campanella@uniroma2.it

Table S1.

Statistically significant (padj <0.05) genera in the three comparisons: OC *vs* PAI-3, OC *vs* PAI-1 and PAI-1 *vs* PAI-3. The ↓ indicates a significant decreased abundance and ↑ indicates a significant increased abundance in the second term of the comparison.

**Genus**	**OC *vs* PAI-3**	**OC *vs* PAI-1**	**PAI-1 *vs* PAI-3**
Haemophilus	↓	↓	-
Neisseria	↓	↓	-
Leptotrichia	↓	↓	-
Granulicatella	↓	↓	-
Gemella	↓	↓	-
Capnocytophaga	↓	↓	-
Saccharibacteria_(TM7)_[G-1]	↓	↓	-
Actinomyces	↓	↓	-
Bergeyella	↓	↓	-
Corynebacterium	↓	↓	-
Rothia	↓	↓	-
Streptococcus	↓	↓	-
Schaalia	↓	↓	-

Lachnoanaerobaculum	↓	↓	-
Lautropia	↓	↓	-
Olsenella	↑	-	-
Cardiobacterium	↓	↓	-
Aggregatibacter	↓	↓	-
Veillonella	↓	↓	-
Pseudoramibacter	↑	-	↑
Dialister	↑	↑	-
Slackia	↑	-	-
Mycoplasma	↓	-	-
Bacteroidaceae_[G-1]	↑	-	-
Porphyromonas	↓	-	-
Saccharibacteria_(TM7)[G-3]	↓	-	-
Bacteroidales_[G-2]	↓	↓	-
Ruminococcaceae_[G-2]	↓	-	-
Kingella	↓	↓	-
Campylobacter	↓	↓	-
Saccharibacteria_(TM7)[G-5]	↓	-	-
Peptostreptococcaceae_[XI][G-6]	↑	↑	-
Bacteroidetes_[G-5]	↓	↓	-
Ruminococcaceae_[G-1]	↓	↓	-
Peptostreptococcaceae_[XI][G-1]	↑	↑	-
Tannerella	↓	-	-
Atopobium	↑	↑	-
Abiotrophia	↓	-	-
Catonella	↓	-	-
Lactobacillus	↑	↑	-

Mogibacterium	↑	-	-
Fusobacterium	↓	↓	-
Absconditabacteria_(SR1)[G-1]	↓	-	-
Stomatobaculum	↓	↓	-
Fretibacterium	↑	↑	-
Anaeroglobus	↑	-	-
Oribacterium	↑	↓	-
Megasphaera	↓	-	-
Lachnospiraceae_[G-3]	↓	-	-
Enterococcus	-	↑	-
Selenomonas	-	↓	-
Pseudomonas	-	↑	-
Paracoccus	-	↑	-
Alloprevotella	-	↓	-
Erysipelotrichaceae_[G-1]	-	↑	-
Eikenella	-	↓	-
Peptostreptococcaceae_[XI][G-3]	-	-	↑
